# Effects of nonpharmacological intervention on sleep quality in hemodialysis patients: A protocol for systematic review and meta-analysis

**DOI:** 10.1097/MD.0000000000026401

**Published:** 2021-07-09

**Authors:** Hui Li, Long Zuo, Siyu Long, Baifei Li

**Affiliations:** Department of Nephrology, The First Affiliated Hospital of Chongqing Medical University, Chongqing, China.

**Keywords:** hemodialysis, meta-analysis, non-pharmacological intervention, protocol, sleep

## Abstract

**Background:**

Nonpharmacological intervention can improve the sleep quality of hemodialysis patients. However, there are many types of nonpharmacological interventions, which makes it difficult to determine the best one. Therefore, this study carried out network meta-analysis to evaluate the effects of nonpharmacological intervention on sleep quality of hemodialysis patients, so as to provide evidence for the selection of the optimal nonpharmacological intervention for the improvement of sleep quality of hemodialysis patients clinically.

**Methods:**

Randomized controlled trials on the effects of nonpharmaceutical interventions on sleep quality in hemodialysis patients were conducted by searching English databases (PubMed, Cochrane Library, EMBASE, and Web of Science) and Chinese databases (Chinese Scientific Journal Database, China National Knowledge Infrastructure Database, Wanfang, and China Biomedical Literature Database) on computer. The retrieval time was from the establishment of the database to May 2021. Literature screening, data extraction, and evaluation of the risk of bias in the included studies were conducted independently by two researchers. Data analysis was performed with STATA14.0 and GEMTC 0.14.3 software.

**Results:**

We will disseminate the findings of this systematic review and meta-analysis via publications in peer-reviewed journals.

**Conclusions:**

This study will provide the best evidence-based evidence to support the effects of non-pharmacological interventions on sleep quality in hemodialysis patients.

**Ethics and dissemination:**

Ethical approval was not required for this study. The systematic review will be published in a peer-reviewed journal, presented at conferences, and shared on social media platforms. This review would be disseminated in a peer-reviewed journal or conference presentations.

**OSF REGISTRATION NUMBER:**

DOI 10.17605/OSF.IO/4BPKT.

## 1 Introduction

Hemodialysis is the main treatment for patients with end-stage renal disease. According to WHO statistics, about 500 million patients suffer from chronic kidney disease in the world, and the rate increases by 7% every year, among which 2% will progress to the stage of end-stage renal disease.^[1,2]^

In recent years, with the progress of science and technology and medical treatment, the hemodialysis technology is gradually improved, which has greatly extended the survival time of end-stage renal disease. However, most hemodialysis patients do not have a high quality of life. Chronic hemodialysis patients experience restless leg syndrome, itchy skin, thirst, depression, and other symptoms, thus leading to sleep disturbances.^[3–5]^

At present, a study has revealed that up to 86.6% of hemodialysis patients have sleep disorders.^[6]^ At present, the main methods for conventional treatment of sleep disorders in hemodialysis patients include psychological treatment, lifestyle adjustment, drug treatment, external treatment of Traditional Chinese Medicine, and so on.^[7–9]^

Western medicine uses sedation, hypnosis, and antipsychotic drugs in treating insomnia of hemodialysis patient commonly, and the side effects are huge. Therefore, it is of great clinical significance to explore non-drug intervention schemes in improving the sleep quality of hemodialysis patients.

Many different types of nonpharmacological therapies have different effects, and there are insufficient direct comparisons on the effectiveness among different nonpharmacological therapies.^[10–15]^ Therefore, this study conducted network meta-analysis to evaluate the effects of different nonpharmacological therapies on sleep quality of hemodialysis patients, so as to provide evidence for the selection of the optimal nonpharmacological intervention plan and improve the sleep quality of hemodialysis patients.

## 2 Methods

### 2.1 Study registration

The protocol of this review was registered in OSF (OSF registration number: DOI 10.17605/OSF.IO/4BPKT). It was reported to follow the statement guidelines of preferred reporting items for systematic reviews and meta-analyses protocol.^[16]^

### 2.2 Inclusion criteria for study selection

1. Study type: Randomized controlled trials and limitation to Chinese and English languages;
2. Participants: Patients who have been on hemodialysis for ≥3 months and are in stable condition;
3. Interventions: The intervention group received non-drug intervention programs, such as aerobic exercise, acupuncture, yoga, massage, and so on. The control group received conventional treatments, including placebo, esazolam, sulozam, and so on.
4. Outcome indicators: Pittsburgh Sleep Quality Index; Richards-Campbell Sleep Questionnaire.

### 2.3 Exclusion criteria

1. Duplicate publications;
2. Incomplete data;
3. Interventions that do not conform to the literature.

### 2.4 Data sources

PubMed, Cochrane Library, EMBASE, and Web of Science, Chinese Scientific Journal Database, China National Knowledge Infrastructure Database, Wanfang, and China Biomedical Literature Database were systematically searched. The time for literature retrieval was set from the establishment of the database until May 2021.

### 2.5 Searching strategy

The details of PubMed's search strategies are illustrated in Table 1, including all search terms, whereas similar search strategies are applied to other electronic databases.

**Table 1:** Search strategy in PubMed database.

Number	Search terms
#1	Renal Dialysis[MeSH]
#2	Dialysis, Extracorporeal[Title/Abstract]
#3	Dialysis, Renal[Title/Abstract]
#4	Extracorporeal Dialysis[Title/Abstract]
#5	Hemodialysis[Title/Abstract]
#6	Dialyses, Extracorporeal[Title/Abstract]
#7	Dialyses, Renal[Title/Abstract]
#8	Extracorporeal Dialyses[Title/Abstract]
#9	Hemodialyses[Title/Abstract]
#10	Renal Dialyses[Title/Abstract]
#11	or/1–10
#12	Treatment[Title/Abstract]
#13	Intervention[Title/Abstract]
#14	Therapy[Title/Abstract]
#15	Management[Title/Abstract]
#16	Rehabilitation[Title/Abstract]
#17	or/12–16
#18	Sleep[MeSH]
#19	Sleep, Slow-Wave[Title/Abstract]
#20	Sleep, Slow Wave[Title/Abstract]
#21	Slow-Wave Sleep[Title/Abstract]
#22	or/18–21
#23	Randomized Controlled Trial[MeSH]
#24	Random^∗^[Title/Abstract]
#25	Clinic trial [Title/Abstract]
#26	or/23–25
#27	#11 and #17 and #22 and #26

### 2.6 Data collection and analysis

#### 2.6.1 Literature screening and data extraction

**Figure 1:**
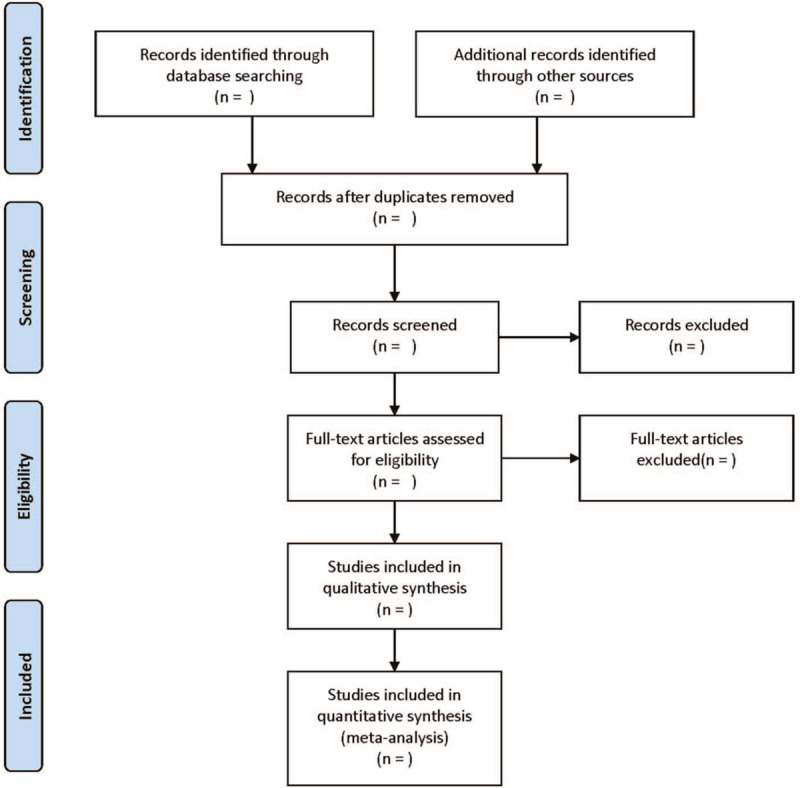
Flow diagram of study selection process.

The screening flow chart of this study is demonstrated in Figure 1. Literature screening and data extraction were conducted independently and cross-checked by 2 researchers. A third researcher will discuss and decide when there is a disagreement. Relevant information was extracted as follows: first author, publication year, sample size, sex, age, course of disease, intervention measures, course of treatment, and outcome indicators.

#### 2.6.2 Assessment of risk of bias

The Cochrane System Evaluation Manual version 5.1.0 randomized controlled trial bias risk assessment tool was used through random sequence generation, allocation concealment, blinding of participants and 7 items, personnel, blinding of outcome assessment, incomplete outcome data, selective reporting, and other bias to assess the quality of the included studies.

#### 2.6.3 Measures of treatment effects

Standard mean difference and 95% confidential interval were pooled.

#### 2.6.4 Management of missing data

If any data are missing, the original data will be requested by email. If the missing data cannot be obtained, the data could be excluded from the study.

#### 2.6.5 Assessment of heterogeneity and data synthesis

Stata14.0 software was used to draw an evidence network map to show the comparison of the intervention measures for each outcome indicator. *χ*^2^ Test was performed to measure the heterogeneity among the direct comparison results, and *I*^2^ was used to measure the heterogeneity. If the data of the included studies displayed no statistical heterogeneity (*I*^2^ < 50%, *P* > .1), the fixed-effect model was used for meta-analysis; on the contrary, random-effects model was adopted for meta-analysis. Meanwhile, GEMTC 0.14.3 software was used to perform mesh meta-analysis based on the Markov Chain-Monte Carlo (MCMC) fitting consistent model under the Bayesian framework. Four chains were used for simulation, and the number of iterations was set as 50,000 (the first 20,000 for annealing and the last 30,000 for sampling). The estimation and inference are carried out under the assumption that MCMC achieves stable convergence state. The stability and consistency of the results were evaluated by adopting the MCMC fitted inconsistency model.

#### 2.6.6 Assessment of reporting biases

“Comparison-adjusted” funnel plot was drawn to evaluate publication bias.

#### 2.6.7 Subgroup analysis

Subgroup analysis would be applied based on the course of treatment and types of sleep quality scales.

#### 2.6.8 Sensitivity analysis

Through the elimination one by one, the sensitivity analysis was performed to test the stability of the results of meta-analysis.

#### 2.6.9 Ethics and dissemination

The content of this article does not involve moral approval or ethical review and would be presented in print or at relevant conferences.

## 3 Discussion

Hemodialysis patients have poor sleep quality, such as insomnia, easy to wake up, poor sleep quality, difficulty to fall asleep after waking, daytime sleepiness, and other sleep problems.^[17,18]^ Sleep quality of hemodialysis patients seriously affects their quality of life, and even affects the survival rate.^[19–21]^ A growing body of evidence suggests that nonpharmacological intervention for primary insomnia responds well with fewer adverse events,^[22–24]^ and it is also a potentially effective way to improve the sleep quality of hemodialysis patients. However, the results of clinical studies on the effects of nonpharmacological interventions on sleep quality in hemodialysis patients are inconsistent. In this study, network meta-analysis was applied to comprehensively summarize and quantify the effects of nonpharmacological intervention on sleep quality of hemodialysis patients, so as to provide assistance for evidence-based clinical decision-making.

## Author contributions

**Conceptualization:** Baifei Li.

**Data curation:** Hui Li, Long Zuo.

**Formal analysis:** Hui Li, Long Zuo.

**Funding acquisition:** Baifei Li.

**Investigation:** Long Zuo.

**Methodology:** Singyu Long.

**Project administration:** Baifei Li.

**Supervision:** Baifei Li.

**Validation:** Long Zuo, Siyu Long.

**Visualization and software:** Long Zuo.

**Visualization:** Siyu Long.

**Writing – original draft:** Hui Li and Baifei Li.

**Writing – review & editing:** Hui Li and Baifei Li.
